# The Effect of Rapid Point-of-Care Respiratory Pathogen Testing on Antibiotic Prescriptions in Acute Infections—A Systematic Review and Meta-analysis of Randomized Controlled Trials

**DOI:** 10.1093/ofid/ofad443

**Published:** 2023-08-18

**Authors:** Ilari Kuitunen, Marjo Renko

**Affiliations:** Department of Pediatrics, University of Eastern Finland, Institute of Clinical Medicine, Kuopio, Finland; Department of Pediatrics, Kuopio University Hospital, Kuopio, Finland; Department of Pediatrics, University of Eastern Finland, Institute of Clinical Medicine, Kuopio, Finland; Department of Pediatrics, Kuopio University Hospital, Kuopio, Finland

**Keywords:** polymerase chain reaction, point-of-care, rapid diagnostics

## Abstract

**Background:**

Rapid point-of-care testing for respiratory pathogens has gained increasing popularity, but its impact on antibiotic consumption is unclear. Thus, the aim of this systematic review and meta-analysis was to estimate the effect of rapid point-of-care testing on antibiotic prescriptions.

**Methods:**

The search for this systematic review with meta-analysis was performed in February 2023. Randomized controlled trials investigating the impact of testing for respiratory pathogens in all-aged patients were included regardless of the comparator. The main outcome was the antibiotic prescription rate. Analyses were stratified by test type, test setting, and patient age. A random-effects Mantel-Haenszel model was used to calculate risk ratios with 95% confidence intervals. Risk of bias was assessed for included studies, and the quality of the evidence was rated according to GRADE.

**Results:**

A total of 754 abstracts were screened, and 10 studies were included in the analysis. Risk of bias was high in 2, low in 4, and had some concerns in 4 studies. Four studies analyzed influenza and respiratory syncytial virus tests, and 6 studies analyzed multiplex (viral and/or bacterial) testing. The prescription rate was 48.2% (496/1029) in the influenza and respiratory syncytial virus test group and 48.7% (540/1109) in the control group (risk ratio [RR], 0.97; 95% CI, 0.92–1.02; moderate-quality evidence). The prescription rate in the multiplex testing group was 54.3% (1554/2859), and it was 57.3% (1336/2326) in the control group (RR, 1.00; 95% CI, 0.96–1.04; moderate-quality evidence). In an age-stratified analysis, the prescription rates showed no evidence of a difference (children: RR, 1.03; 95% CI, 0.81–1.30; adults: RR, 0.98; 95% CI, 0.96–1.01; very low- and moderate-quality evidence).

**Conclusions:**

We found moderate-quality evidence that rapid point-of-care testing for respiratory pathogens does not decrease the antibiotic prescription rate.

Interventions to reduce antibiotic prescriptions are needed as overuse of antibiotics has been declared one of the key issues in medicine [1–3]. Antibiotics cause both short-term and long-term harms [4], and overuse worsens resistance issues globally [1]. We know that the majority of respiratory tract infections are viral [5, 6], and most resolve without antibiotics [7]. However, antibiotic treatment is still needed for bacterial diseases. In some cases (eg, pneumonia), the separation of viral and bacterial etiology is controversial, and thus all cases are treated with antibiotics [8].

Point-of-care C-reactive protein testing has been shown to reduce antibiotic prescriptions [9]. Introduction of fast point-of-care tests for respiratory microbes has raised hope that recognition of viruses in patients with respiratory tract infections could lead to lower prescription rates [10]. Reductions in antibiotic prescriptions have been seen in observational studies and pre–post intervention studies [11, 12]. However, recent randomized studies have not shown a meaningful impact of rapid testing on reducing antibiotic prescription rates [13–15].

The aim of this systematic review and meta-analysis was to analyze the impact of rapid point-of-care microbial testing of respiratory pathogens on antibiotic prescriptions in acute infections.

## METHODS

### Search Process

We searched the PubMed, Scopus, and Web of Science databases on February 15, 2023. The complete search strategy is provided in the Supplementary Data. Search results were then uploaded to COVIDence (Veritas Health Innovation, Melbourne, Australia) for screening. Two authors independently screened the abstracts and full texts. In cases of disagreement, a mutual consensus was reached by discussion.

### Inclusion and Exclusion Criteria

We included randomized controlled studies that compared rapid point-of-care viral and/or bacterial polymerase chain reaction (PCR) testing regardless of the blinding and comparator. We excluded all observational studies. Studies that did not present any original data (reviews, editorials, etc.) were excluded. Non-English-language reports were also excluded.

### Outcome Measures

Our main outcome was the antibiotic prescription rate. We defined antibiotic prescription as any antibiotic given for any indication during the study period. The secondary outcome was the antibiotic treatment duration in days. Stratified analyses between the test type (influenza + respiratory syncytial virus [RSV] and/or multiplex test), test setting (outpatients or inpatients), and age of the patients (children or adults) were conducted. “Multiplex test” means that a single sample is analyzed for several viruses and/or bacteria using the PCR method.

### Data Extraction

The following data were extracted by 1 author from each included study to a predesigned Excel worksheet: authors, journal, country, setting, main outcomes, secondary outcomes, number of participants in each group, number of antibiotic prescriptions, antibiotic treatment duration.

### Statistics

This review was conducted according to the guidelines in the *Cochrane Handbook of Systematic Reviews* [16]. Studies were pooled together in a meta-analysis. A random-effects model was chosen due to expected heterogeneity between the studies (patient population and study settings). Risk ratios with 95% confidence intervals were calculated with the Mantel-Haenszel test. Mean difference (MD) was calculated using the random-effects inverse variance method for continuous outcomes. Sensitivity analyses for comparisons were performed in which the studies with high risk of bias were excluded. Review Manager, version 5.4.1, was used for all statistical analyses. Publication bias was estimated from funnel plots visually.

Risk of bias was assessed according to the Cochrane risk of bias 2.0 tool [17]. Risk of bias is presented for each individual study and as a summary plot per assessed domains. Figures were generated by robvis shinyapp [18]. Quality of evidence for the main outcomes was assessed according to the Grading of Recommendations, Assessment, Development and Evaluations (GRADE) framework [19]. This study has been reported according to the Preferred Reporting Items in Systematic Reviews and Meta-Analyses 2020 (PRISMA) guidelines; the checklist can be found in the Supplementary Data [20].

### Protocol Registration

The study protocol was registered with PROSPERO.

## RESULTS

**Figure 1.**
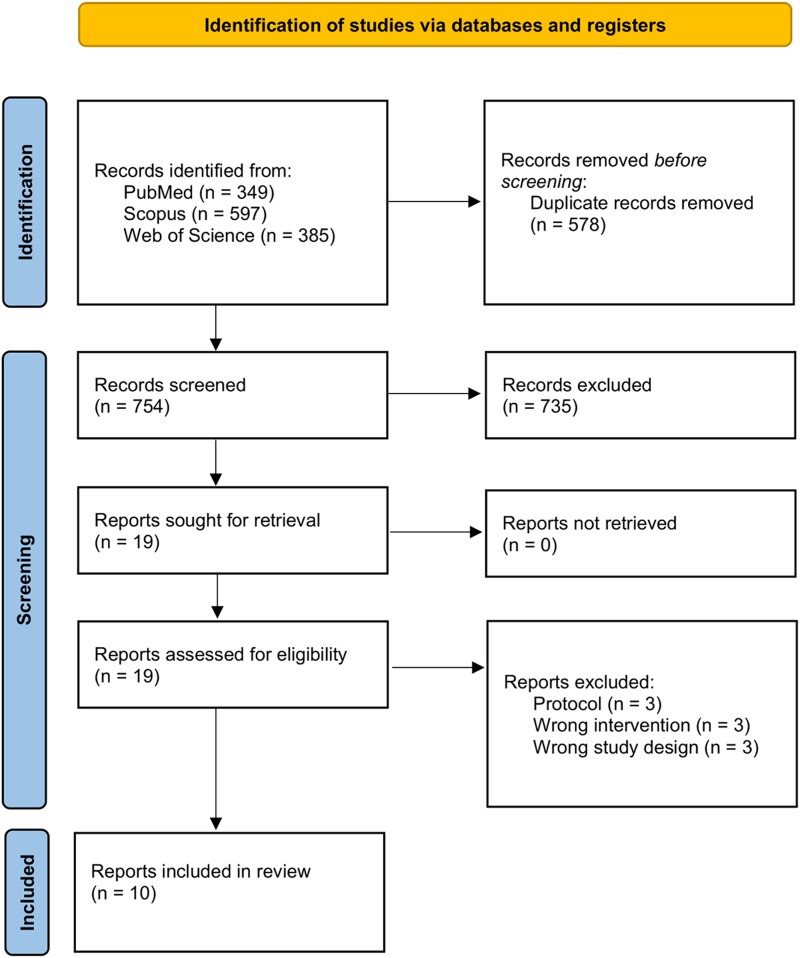
PRISMA flowchart of the study selection process. Abbreviation: PRISMA, Preferred Reporting Items in Systematic Reviews and Meta-Analyses.

Our search yielded a total of 754 unique items. After screening of titles and abstracts, we further assessed 19 studies and included 10 studies for analysis (Figure 1) [13–15, 21–27]. Five studies were conducted in Europe, 4 in Northern America, and 1 in China (Table 1). None of the studies were blinded. The control was either routine clinical care or delayed laboratory microbial testing. Six studies analyzed multiplex arrays, and 4 studies analyzed influenza or influenza/RSV arrays.

**Table 1.** Characteristics of the Included Studies

Study	Country	Study Period	Blinding	Intervention	Control	Main Outcome	Inclusion Criteria	Exclusion Criteria
Andrade et al 2023 [21]	USA	2018–2022	No	Point-of-care flu/RSV array	Usual care	Antibiotic and antiviral prescription rate	Influenza-like illness	COVID-19 positive
Andrews et al 2017 [22]	UK	2015	No	Point-of-care multiplex array	Laboratory PCR for viruses and cultures for bacteria	Length of stay	Respiratory tract infection	Co-occurring/suspected other bacterial infection
Bibby et al 2022 [25]	Canada	2020	No	Point-of-care flu/RSV array	Laboratory PCR	Length of stay	Not specified	Not specified
Brensdish et al 2017 [24]	UK	2014–2016	No	Point-of-care multiplex array	Laboratory PCR for viruses and cultures for bacteria	Antibiotic prescription rate	Acute respiratory illness	Palliative care patients
Clark et al 2021	UK	2017–2019	No	Point-of-care multiplex array	Routine clinical care	Antiviral prescription rate	Acute respiratory illness	Palliative care patients, or a patient was already included once
Mattila et al 2022 [14]	Finland	2019–2020	No	Point-of-care multiplex array	Routine clinical care	Antibiotic prescription rate	Fever and/or respiratory symptoms	Need for immediate transfer to intensive care unit upon arrival
Rao et al 2021 [15]	USA	2018–2019	No	Point-of-care multiplex array	Routine clinical care	Antibiotic prescription rate	Influenza-like illness	Symptoms for >14 d, nurse only visit, previous
Saarela et al 2020 [13]	Finland	2014–2015	No	Point-of-care multiplex array	Delayed viral testing in laboratory	Length of stay and antibiotic prescription rate	Any respiratory infection symptom and fever or chest pain or poor general condition for an unknown reason	No exclusion criteria were applied
Schechter-Perkins et al 2019 [26]	USA	2017	No	Point-of-care flu array	Laboratory flu antigen or PCR test	Length of stay and antibiotic prescription rate	Influenza-like illness	Multiplex test array ordered, influenza test results already known upon arrival
Shengchan et al 2019 [27]	China	2018	No	Point-of-care multiplex array	Laboratory PCR for viruses	Intravenous antibiotic duration	Lower respiratory tract infection, hospitalized patient	Pregnant, hospital-acquired pneumonia, lung tuberculosis; HIV, hematological cancer, or solid tumor treated with chemotherapy or radiotherapy in the previous 3 mo, organ or bone marrow transplantation, splenectomy, or autoimmune diseases

Abbreviations: COVID-19, coronavirus disease 2019; PCR, polymerase chain reaction; RSV, respiratory syncytial virus.

### Risk of Bias

**Figure 2.**
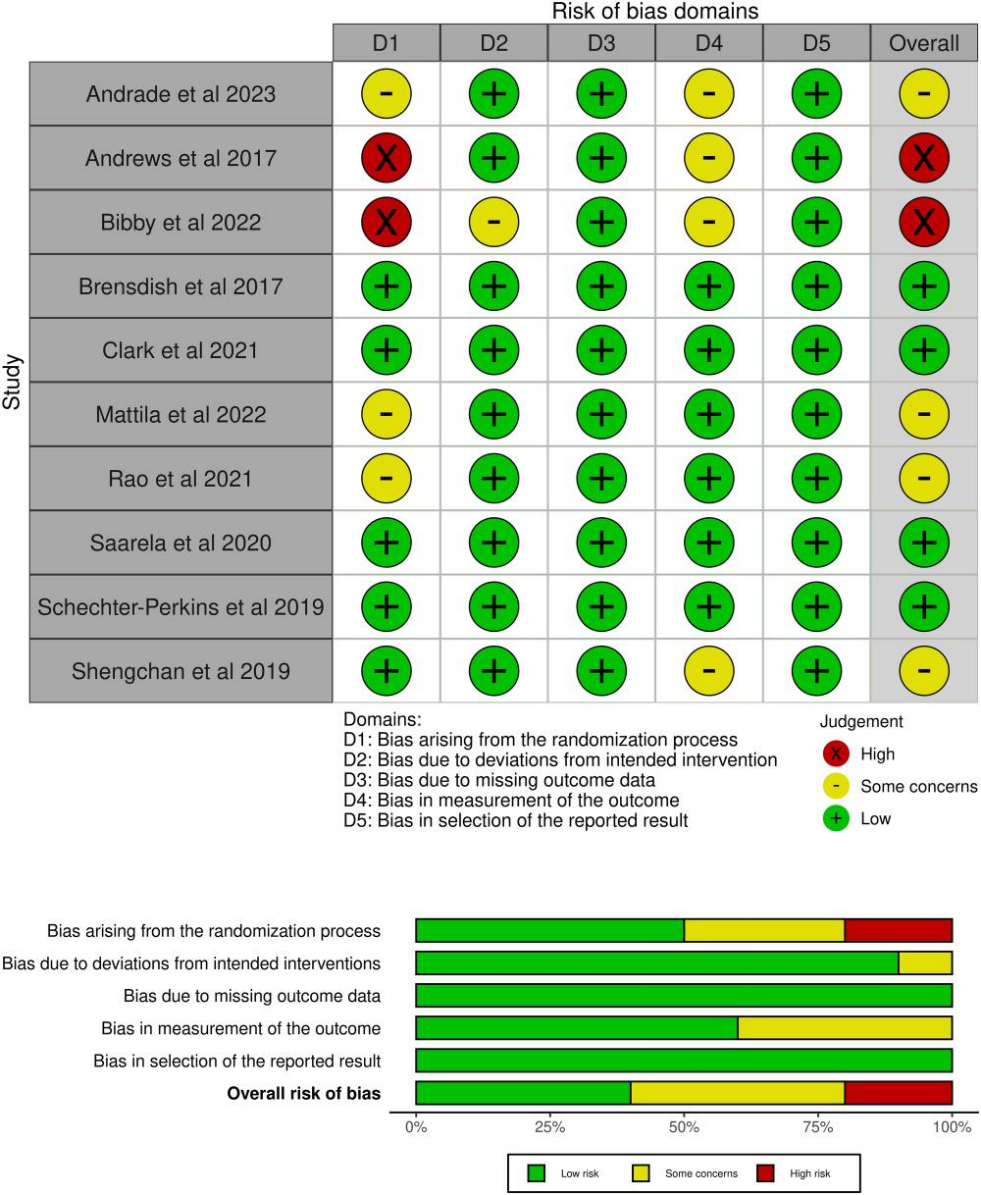
Risk of bias analyzed in 5 domains and overall according to the Cochrane Risk of Bias 2.0 tool.

The overall risk of bias was high in 2 studies, had some concerns in 4 studies, and was low in 4 studies (Figure 2). Most issues came from the bias arising from the randomization process or in the measurement of the outcome (Figure 2).

### Antibiotic Prescription Rate

**Figure 3.**
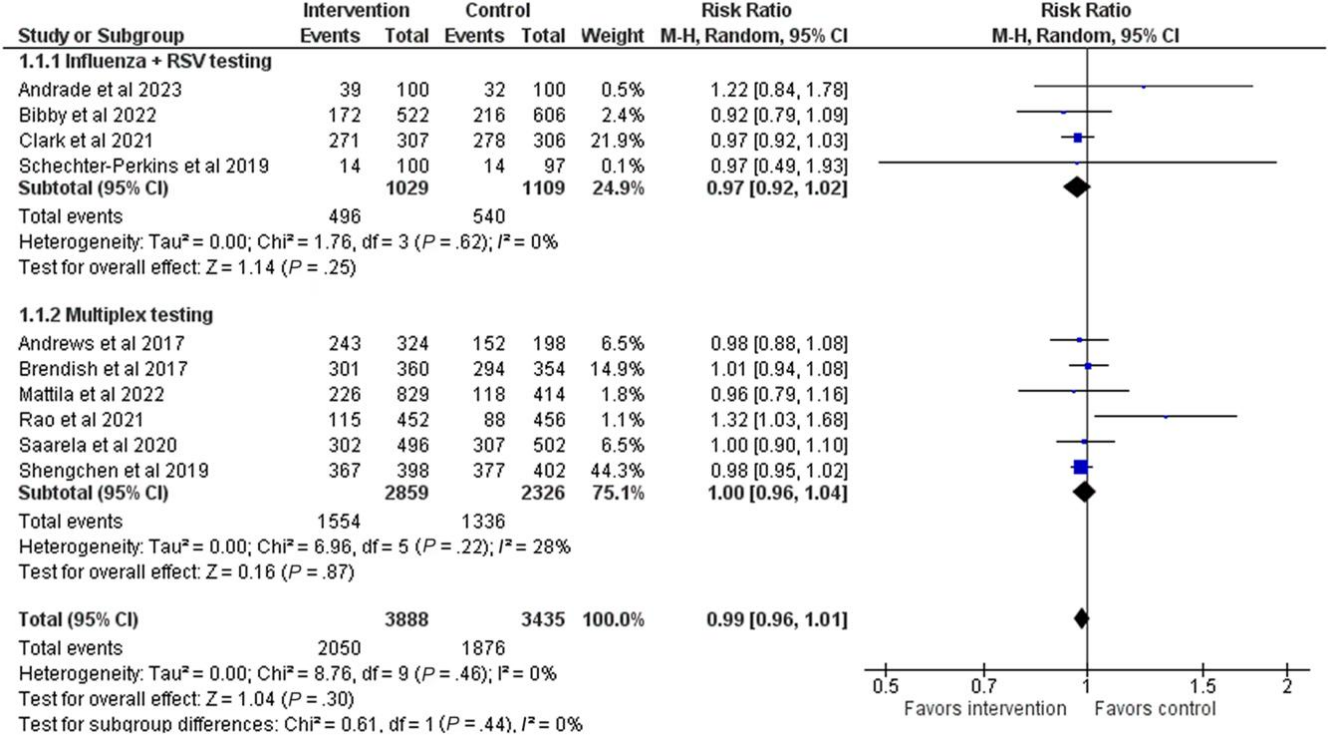
Forest plot of antibiotic prescriptions stratified by the type of rapid virus testing (influenza + RSV or multiplex test). Abbreviation: RSV, respiratory syncytial virus.

Antibiotic prescription rate was assessed in all 10 studies, with 7323 patients. The prescription rate was 52.7% in the intervention group and 54.6% in the control group (risk ratio [RR], 0.99; 95% CI, 0.96–1.01) (Figure 3). We ranked the quality of evidence as moderate (Table 2). In the influenza + RSV test group, the RR was 0.97 (95% CI, 0.92–1.02; 4 studies), and in the multiplex group the RR was 1.00 (95% CI, 0.96–1.04; 6 studies) (Figure 2). An additional sensitivity analysis that excluded high–risk of bias studies did not change the effect estimates (Supplementary Figure 1). Publication bias was not detected (Supplementary Figure 2).

**Table 2.** Summary of Findings Table and GRADE Assessment for Each Outcome

Outcome	Absolute Effect		Relative Effect	No. of Patients (No. of Studies)	GRADE
	Intervention Group	Control Group			
Antibiotic prescription rate	53 per 100	55 per 100	RR, 0.99 (95% CI, 0.96–1.01)	7323 (10)	Moderate^a^
Test type					
Influenza or influenza and RSV	48 per 100	49 per 100	RR, 0.97 (95% CI, 0.92–1.02)	2138 (4)	Moderate^a^
Multiplex test	54 per 100	57 per 100	RR, 1.00 (95% CI, 0.96–1.04)	5185 (6)	Moderate^a^
Study setting					
Outpatients	19 per 100	14 per 100	RR, 1.26 (95% CI, 0.97–1.64)	1543 (4)	Low^b^
Inpatients	74 per 100	77 per 100	RR, 0.97 (95% CI, 0.95–1.00)	2825 (6)	Low^b^
Patient age					
Children	26 per 100	25 per 100	RR, 1.03 (95% CI, 0.81–1.30)	2857 (3)	Very low^c^
Adults	79 per 100	80 per 100	RR, 0.98 (95% CI, 0.96–1.01)	3647 (5)	Moderate^a^
Antibiotic treatment duration					
Inpatients	-	-	MD, −0.59 (95% CI, −1.32 to 0.13) d	1327 (3)	Very low^d^
All patients	-	-	MD, −0.06 (95% CI, −1.33 to 1.21) d	1234 (2)	Very low^d^

Abbreviations: GRADE, Grading of Recommendations, Assessment, Development and Evaluations; RR, risk ratio; RSV, respiratory syncytial virus.

a Downgraded due to risk of bias.

b Downgraded due to imprecision and risk of bias.

c Downgraded twice due to risk of bias and once due to imprecision.

d Downgraded due to risk of bias, imprecision, and inconsistency.

**Figure 4.**
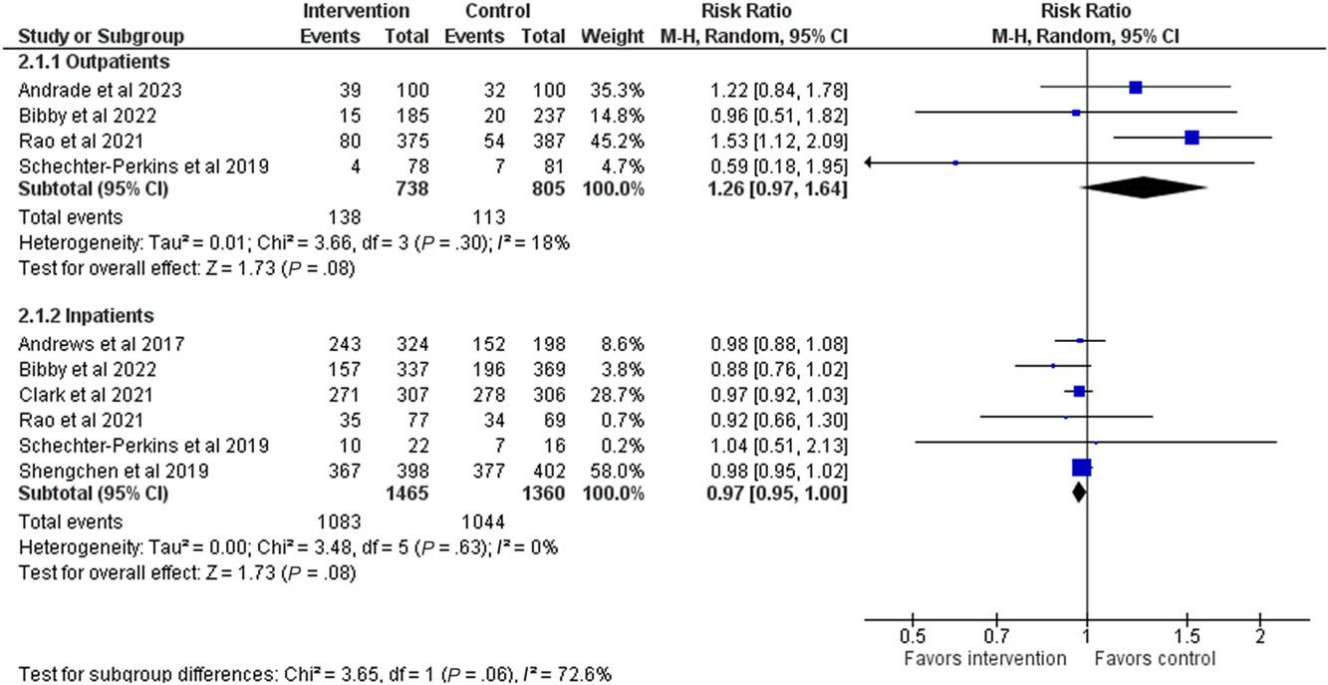
Forest plot of antibiotic prescriptions stratified between outpatients and inpatients.

In outpatients, the antibiotic prescription rate was 18.7% (138/789) in the testing group and 14.0% (113/805) in the control group (RR, 1.26; 95% CI, 0.97–1.64; 4 studies) (Figure 4). The quality of evidence was ranked as low (Table 2). Six studies focused on inpatients, and the prescription rate was 73.9% in the testing group and 76.8% in the control group (RR, 0.97; 95% CI, 0.95–1.00) (Figure 4). The quality of evidence was ranked as low (Table 2). A sensitivity analyses (high–risk of bias studies excluded) did not change the estimates (Supplementary Figure 3), and we did not detect publication bias (Supplementary Figure 4).

**Figure 5.**
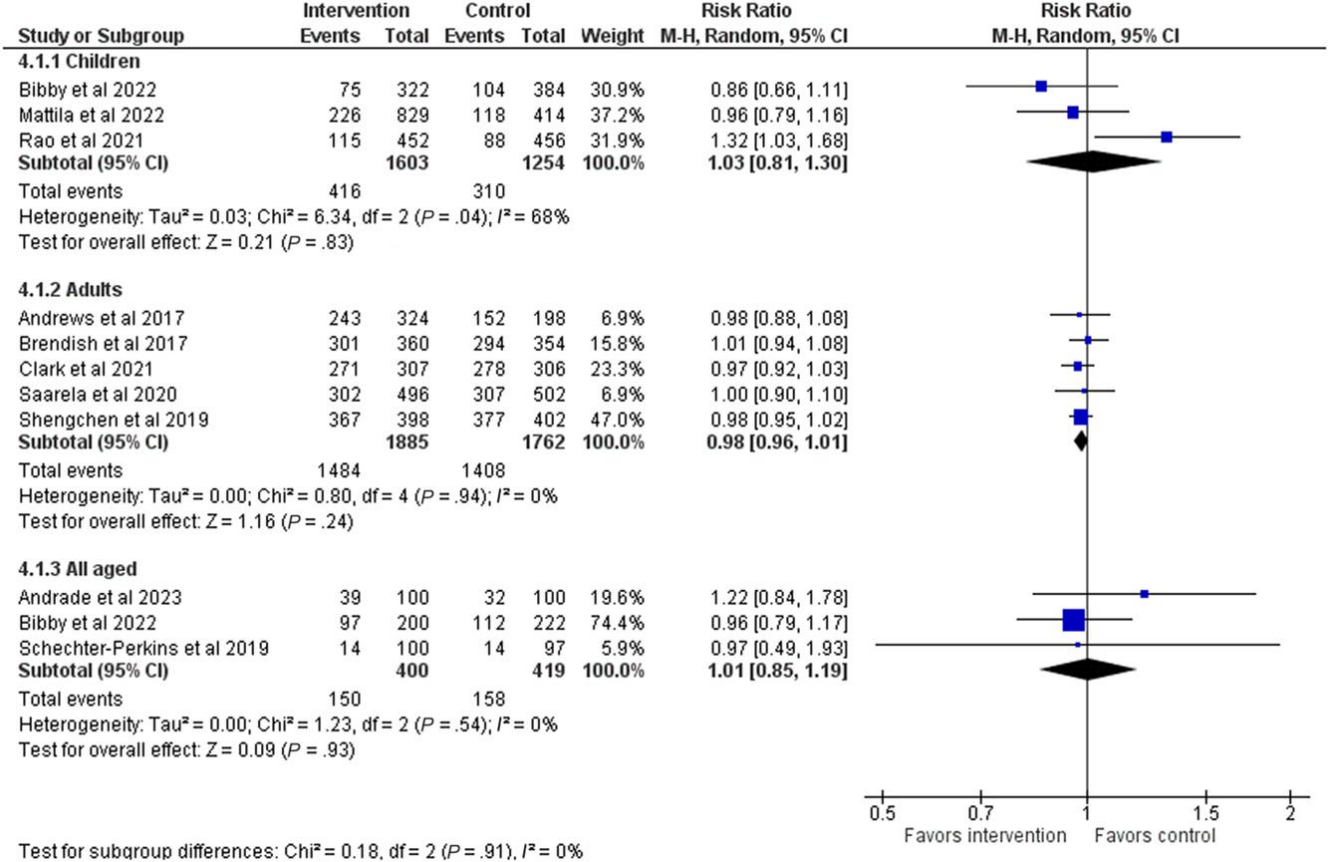
Age-stratified forest plot of antibiotic prescriptions.

Three studies with 1603 patients analyzed the antibiotic prescription rate in children, and the rate was 25.9% (416/1603) in the testing group and 24.7% (310/1254) in the control group (RR, 1.03; 95% CI, 0.81–1.30) (Figure 5). Five studies were conducted in adults only. The antibiotic prescription rate was 78.7% in the testing group and 80.0% in the control group (RR, 0.98; 95% CI, 0.96–1.01) (Figure 5). The quality of evidence was ranked as moderate (Table 2). Additional sensitivity analyses (high–risk of bias studies excluded) did not result in a notable change in the estimate (Supplementary Figure 5). A funnel plot did not show signs of publication bias (Supplementary Figure 6).

### Antibiotic Treatment Duration

**Figure 6.**
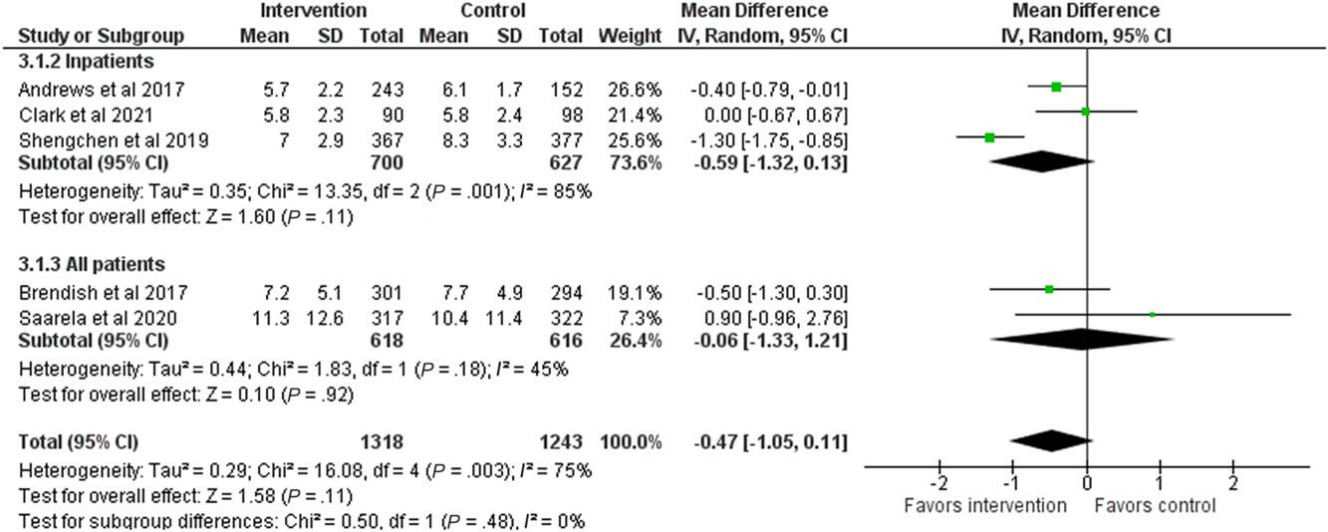
Forest plot of antibiotic treatment duration stratified between inpatients and outpatients.

Five studies with 2561 patients analyzed antibiotic treatment duration, and we found low-quality evidence that antibiotic treatment duration was not shorter in the intervention group (mean difference [MD], −0.47 days; 95% CI, −1.05 to 0.11 days) (Figure 6, Table 2). In a subgroup analysis, the treatment duration among inpatients (MD, −0.59 days; 95% CI, −1.32 to 0.13 days; very low-quality evidence) and both inpatients and outpatients (MD, −0.06 days; 95% CI, −1.33 to 1.21 days; very low-quality evidence) was not shorter in the intervention group than in the control group (Figure 6, Table 2). A sensitivity analysis that excluded high–risk of bias studies did not notably change the estimates (Supplementary Figure 7). We did not detect publication bias (Supplementary Figure 8).

## DISCUSSION

Based on the findings of this review, rapid point-of-care pathogen testing in acute respiratory infections does not reduce antibiotic prescriptions or shorten treatment duration. Additional analyses based on the study setting and patient age did not show benefit of rapid pathogen testing in any of these subgroups.

The findings of this study are consistent with the literature as all previously published randomized trials (also included in this review) have failed to show a reduction in antibiotic prescription rates. The reason for this is most likely the fact that, despite a viral pathogen finding, the decision to prescribe antibiotics comes from the clinical ensemble, where other laboratory parameters, such as C-reactive protein and white blood cell count, also play an important role. In addition to these considerations, there are also several nonmedical factors influencing the antibiotic prescription decision, such as patient preferences and physician attitudes [28, 29]. These previously mentioned factors most likely combine to the effect that antibiotics are prescribed, although in adults, it is known that coinfections of viruses and bacteria are rarer than in children [30].

Hypothetically the beneficial impact of rapid viral testing would especially be seen in children, as children have a very high burden of viral infections [6]. However, in subgroup analyses, the results were similar in children and adults. Many children with viruses detected have coexisting bacterial conditions, as for example based on previous literature acute otitis media is detected in up to 50% of children suffering from upper respiratory tract infections [31]. Furthermore, children have been reported to have a higher test positivity rate for respiratory viruses than adults when they have respiratory tract symptoms [32]. Interestingly, the included studies had higher antibiotic prescription rates in outpatient setting. This was not explained by the higher detection rates of atypical bacteria (eg, *Bordetella pertussis, Chlamydia pneumoniae,* or *Mycoplasma pneumonia*) as 3 of the outpatient studies used influenza and RSV testing [21, 25, 26]. The only study that used multiplex testing had similar rates of atypical bacteria detections between the control and intervention groups [15]. Thus the exact reason for the higher prescription rate in outpatients remains unknown.

Future studies are needed to better guide antibiotic prescriptions and to reduce the burden of antibiotic treatment, especially in children, who are more susceptible to antibiotic-related short-term and long-term harms. A combination study of classic laboratory parameters, pathogen findings, and some novel biomarkers or tests could provide an algorithm-based approach to antibiotic prescriptions. Introduction of rapid quantitative analyses of viral loads in the respiratory tract may also help to predict the actual importance of microbial findings [33]. However, as always, a pragmatic approach is needed in future trials.

### Strengths and Limitations

Our main strength is that we did not have any notable protocol deviations. Most of the limitations came from the quality and heterogeneity of the included studies. Risk of bias rose mostly from the lack of blinding or improper randomization processes. Heterogeneity between the study settings, interventions, and comparators was clear and caused concerns. We aimed to control for this by conducting subgroup and sensitivity analyses, but the results remained similar in all subgroups. Due to these factors, the quality of evidence remained between very low and moderate.

## CONCLUSIONS

Our systematic review found moderate-quality evidence that rapid point-of-care testing for respiratory pathogens does not reduce antibiotic prescription rates. The antibiotic prescription rate was even higher in the outpatient setting in the point-of-care testing group. Further strategies and studies are needed to provide evidence-based interventions for antibiotic prescription reduction.

## Supplementary Material

ofad443_Supplementary_Data

## Supplementary Data

Supplementary materials are available at *Open Forum Infectious Diseases* online. Consisting of data provided by the authors to benefit the reader, the posted materials are not copyedited and are the sole responsibility of the authors, so questions or comments should be addressed to the corresponding author.
