# Supplementary material for: The Effect of Rapid Point-of-Care Respiratory Pathogen Testing on Antibiotic Prescriptions in Acute Infections—A Systematic Review and Meta-analysis of Randomized Controlled Trials
Source: Open Forum Infect Dis. 2023 Aug 18;10(9):ofad443. doi: 10.1093/ofid/ofad443 (PMC10472488; doi:10.1093/ofid/ofad443)

**Supplementary materials**

Complete search strategy

Search was performed on February 15^th^ 2023 in PubMed, Scopus and Web of Science databases.

PubMed: ("rapid viral diagnosis"[All Fields] OR "point of care"[All Fields]) AND "respiratory"[All Fields]) AND ("antibiotic*"[All Fields] OR "antimicrobial*"[All Fields])

SCOPUS: TITLE-ABS-KEY ( ( "rapid viral diagnosis" OR "point of care" ) AND respiratory AND ( antibiotic* OR antimicrobial* ) )

Web Of Science: ( ( "rapid viral diagnosis" OR "point of care" ) AND respiratory AND ( antibiotic* OR antimicrobial* ) ) [All Fields]

Figure S1 Sensitivity analysis for Figure 3. High risk of bias studies omitted.


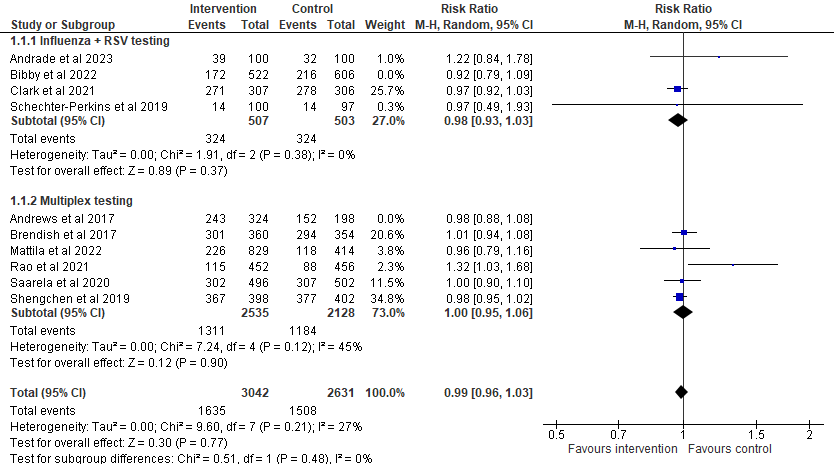


Figure S2 funnel plot of publication bias in the analysis made for figure 3.


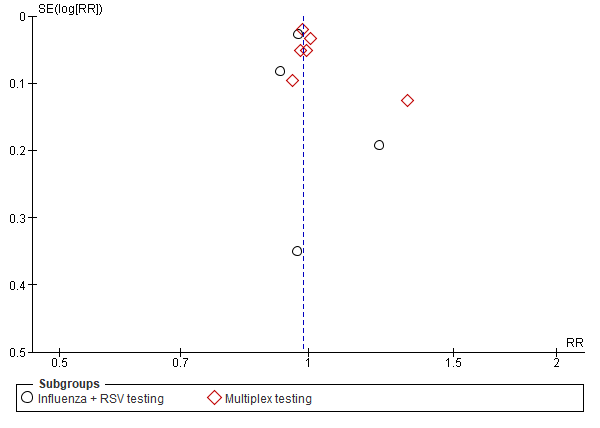


Figure S3 Sensitivity analysis of figure 4, high risk of bias studies excluded.


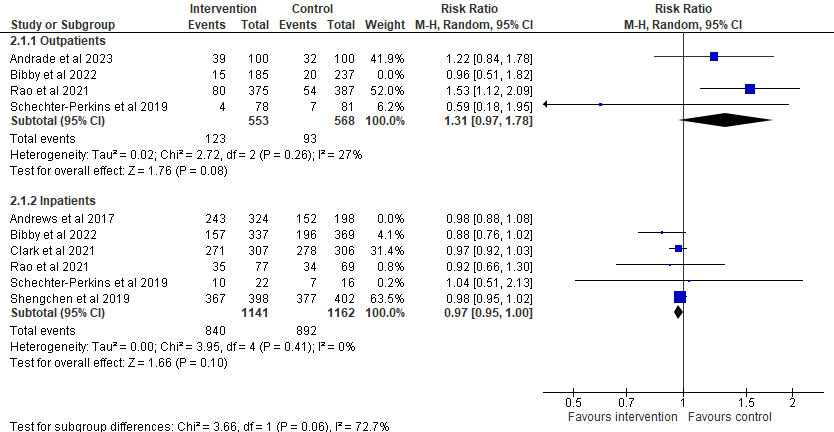


Figure S4 funnel plot of Figure 4


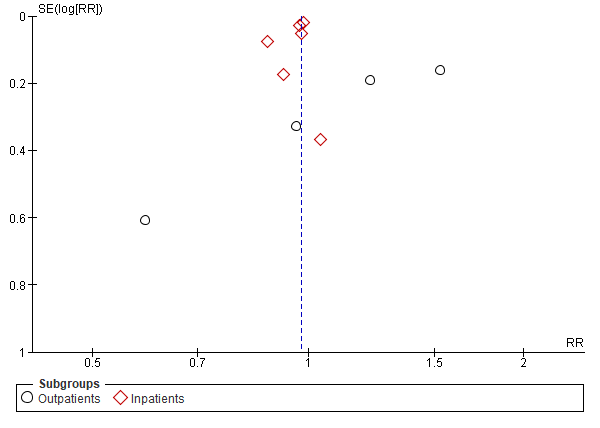


Figure S5 Sensitivity analysis of Figure 5, high risk of bias studies excluded


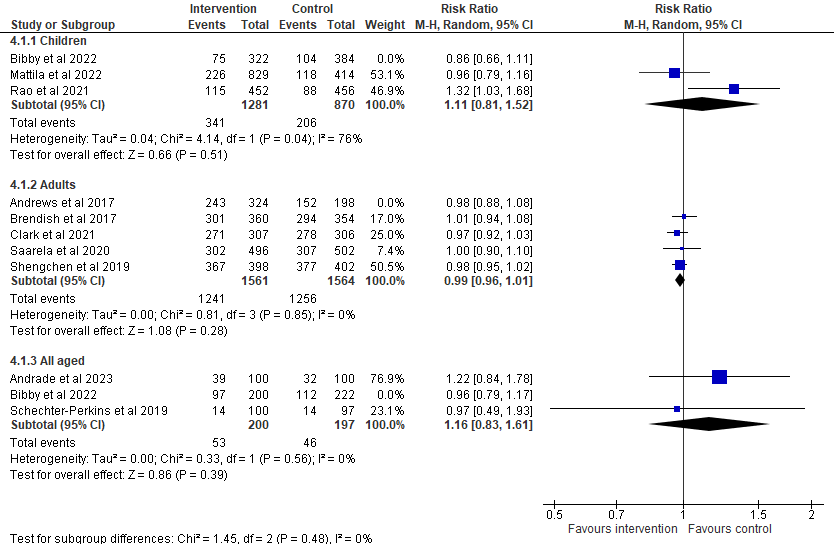


Figure S6 funnel plot


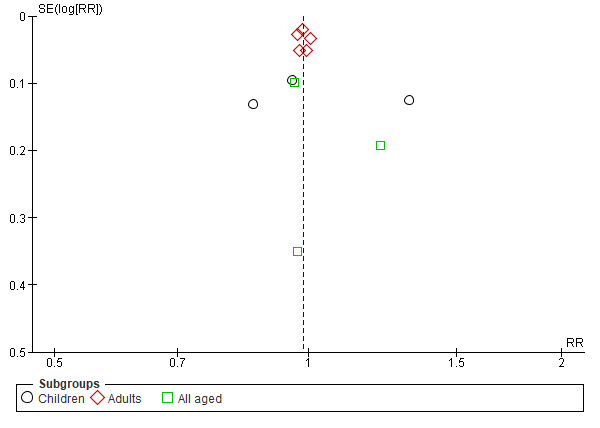


Figure S7 Sensitivity analysis of figure 6, high risk of bias studies excluded.


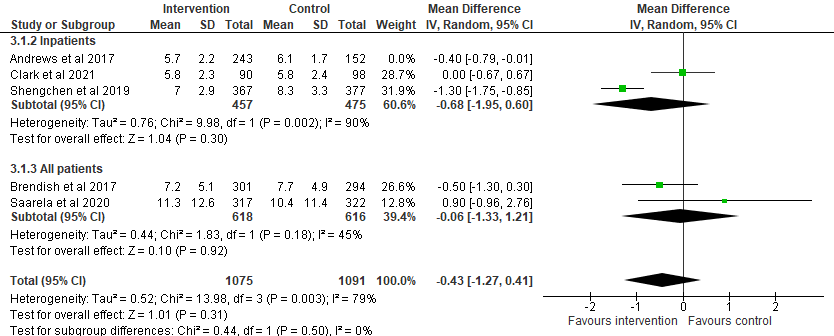


Figure S8 Funnel plot of Figure 6


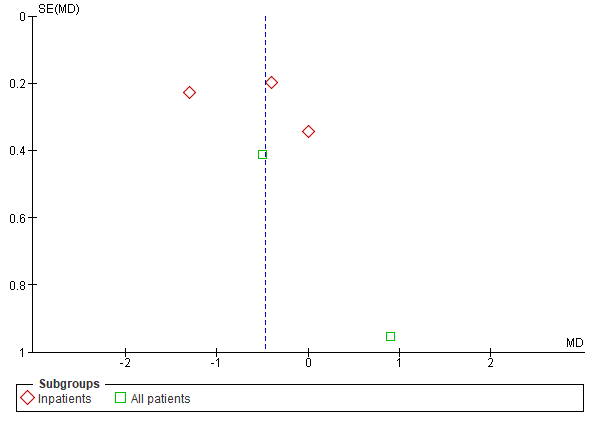

Supplement: ofad443_Supplementary_Data [file ofad443_supplementary_data.docx]
